# Bacterial ribosome heterogeneity facilitates rapid response to stress

**DOI:** 10.1128/jb.00058-25

**Published:** 2025-06-03

**Authors:** Yi-Lin Shen, Lei Xu, Ying Zhou, Bang-Ce Ye

**Affiliations:** 1 Laboratory of Biosystems and Microanalysis, State Key Laboratory of Bioreactor Engineering, East China University of Science and Technology 47860, Shanghai, China; Geisel School of Medicine at Dartmouth, Hanover, New Hampshire, USA

**Keywords:** ribosome heterogeneity, rapid response, post-translational modification

## Abstract

Bacteria live under constant pressure from external signals, necessitating a rapid capacity to reprogram their metabolism. The ribosome, once considered a uniform and static entity, is now recognized for its compositional heterogeneity. Despite its prevalence, the role of this heterogeneity in regulating bacterial translation remains incompletely understood. This review explores how ribosomal heterogeneity may serve as a conserved mechanism for fine-tuning gene expression, enabling swift adjustments to environmental stress. We present recent findings on the regulatory potential of ribosome heterogeneity and its broader implications for bacterial adaptation, pathogenesis, and the development of novel antimicrobial strategies.

## INTRODUCTION

Bacteria are highly adaptable organisms capable of thriving in a wide range of environments and colonizing diverse ecological niches. This remarkable versatility stems from a tightly regulated metabolic network (1), orchestrated by complex feedback mechanisms that allow bacteria to swiftly respond to fluctuating environmental conditions. In their adaptive response to stress, bacteria often target the protein translation process, offering a rapid means of gene expression regulation that extends beyond transcriptional control. Recent advancements in quantitative proteomics and RNA-Seq technologies have provided valuable insights into bacterial stress responses (2–8). While these studies have illuminated transcriptional regulation, the precise interplay between transcriptomic, proteomic, and metabolomic changes remains obscure. Several mechanisms contribute to this fine-tuning (9), yet the specific role of translation modulation remains poorly understood.

**Fig 1:**
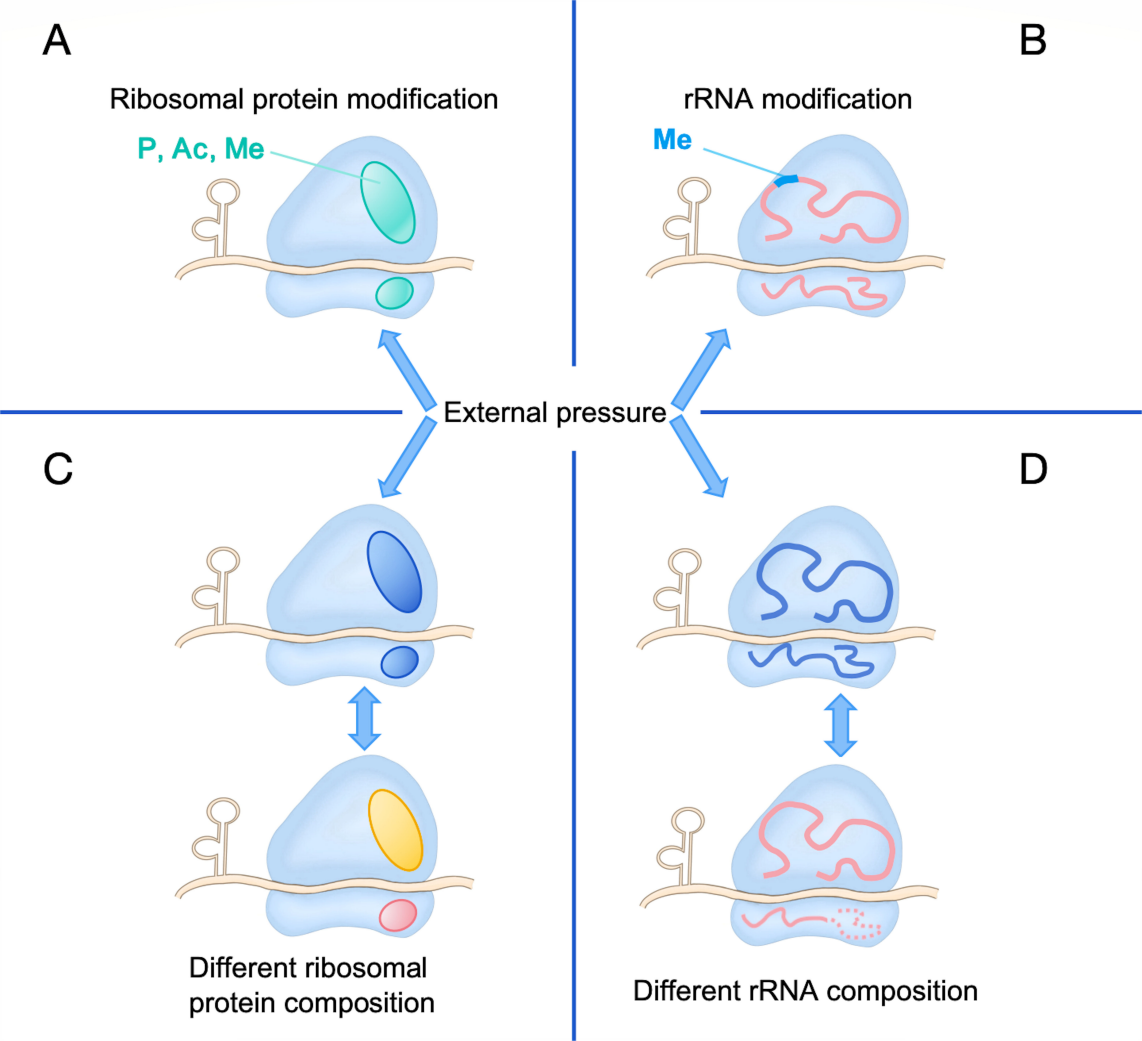
Four types of ribosome heterogeneity. Ribosome heterogeneity can be generated through multiple mechanisms, including chemical modifications, compositional variations, and dynamic interactions with related factors, which collectively fine-tune translation efficiency and specificity. (**A**) Chemical modification of RPs. Chemical modifications (phosphorylation, acetylation, methylation) on specific RPs can alter the function of ribosomes. (**B**) Chemical modification of rRNA. rRNA-specific sites undergo specific modification (methylation). (**C**) Changes in the composition of ribosomal proteins. (**D**) Diversity of rRNA composition.

The translation of the mRNA-encoded genetic information into proteins is controlled by the ribosome, a highly efficient molecular machine, enabling cells to rapidly and transiently respond to a wide range of stimuli and environmental changes, thereby facilitating swift adaptation in growth. Historically, the bacterial ribosome was thought to be composed of a fixed set of ribosomal proteins (RPs) and ribosomal RNA (rRNA), ensuring precise translation (10–12). However, accumulating evidence now points to the existence of heterogeneous ribosomal subunits that exhibit variability in their RP or rRNA components. Heterogeneity is defined as the diversity or variability existing in a biological system, including compositional differences, structural differences, and functional differences, with non-static characteristics that change with time and environment. Generalized ribosome heterogeneity includes differences in RP composition, rRNA diversity, chemical modification of RPs and rRNA (13, 14), and the activity of ribosome-associated factors (Fig. 1), which is one of the important mechanisms affecting protein translation (15). These variations allow the ribosome to modulate the translational program in response to environmental changes (16–20). Ribosome heterogeneity has been implicated in various physiological processes and diseases in eukaryotic and mammalian cells. Notably, the altered content of ribosomes and its influence on gene regulation offers a novel perspective for understanding the role of ribosomes in bacterial adaptability (21, 22).

In response to these developments, an increasing number of studies have explored how bacteria, including pathogens, utilize the adaptive “specialized ribosome” strategy to thrive in challenging environments and ensure survival. Specialized ribosomes specifically refer to a subset of ribosomes with distinct compositional or functional properties, which enable selective translation of specific mRNAs and ribosomes with specific functions. Unlike canonical ribosomes with a standard composition and a broad mRNA translation role, specialized ribosomes are usually actively induced by specific stresses or signaling pathways. Additionally, it has highly specialized functions, such as only translating cleaved mRNAs, and can be reversibly repaired to canonical ribosomes when external stress disappears. This review will specifically examine the translational regulatory mechanisms associated with “stress-mediated ribosomal heterogeneity,” with a focus on how these mechanisms offer novel insights into pathogen-host interactions and potential therapeutic targets for infection control.

### The differential composition of ribosomal proteins and RNAs reshapes the proteome under stress

While the importance of accurate and faithful translation is well-established, emerging evidence indicates that bacterial ribosomes can dynamically reprogram translation, enabling rapid proteome reshaping in response to stress and promoting bacterial survival.

Leaderless translation, mediated by specialized ribosomes, is a critical component of the adaptive strategies utilized by various bacterial species (23). In *Escherichia coli*, two primary mechanisms for generating these specialized ribosomes have been identified: one involves the loss of specific ribosomal proteins, while the other results in structural alterations of the 16S rRNA. Both mechanisms can be triggered by stress conditions, such as antibiotics (24) or bacterial toxins (25). For instance, mutations in *rpsD*, which encodes ribosomal protein S4, have been shown to enhance resistance to oxidative stress and heat in both *E. coli* and *Salmonella enterica* serovar Typhimurium (26–29). Aminoglycoside antibiotics provide another example of stress-induced translation regulation. These antibiotics inhibit the formation of the translation initiation complex by binding to the 30S subunit, disrupting mRNA interaction, and inducing the dissociation of P-site-bound fMet-tRNA^fMet^. Additionally, antibiotics also promote the formation of 61S ribosomes in *E. coli* both *in vitro* and *in vivo* (30). These specialized ribosomes, which lack several proteins such as bS1 and bS21, exhibit structural modifications in the 16S rRNA and exhibit enhanced ability to selectively translate leaderless mRNAs (24). Translation can also be regulated through the MazEF toxin-antitoxin system, which is activated under stress and plays a critical role in bacterial survival and persistence. MazEF system comprises two key components: MazF, a stable toxin that functions as an mRNA ribonuclease to cleave cellular mRNA and block protein synthesis, and MazE, a labile antitoxin that binds and neutralizes MazF. Under stress conditions, such as nutrient starvation, antibiotic exposure, or DNA damage, the unstable antitoxin is degraded, leading to the activation of MazF, which inhibits translation by cleaving both mRNA and rRNA (31, 32). The MazF system specifically targets the 16S rRNA, cleaving the 3´-terminal 43 nucleotides, including the anti-Shine-Dalgarno (a-SD) sequence. This cleavage results in the formation of 70S^Δ43^ stress ribosomes, which selectively translate newly generated leaderless mRNAs. Recent studies have further demonstrated the reversibility of ribosome heterogeneity to recycle the modified ribosomes upon stress relief. By removing the 3´-16S rRNA, MazF generates specialized ribosomes to selectively translate mRNAs likewise processed by MazF, while RNA ligase RtcB catalyzes the re-ligation of the truncated 16S rRNA present in specialized ribosomes (31).

Furthermore, the identification of mutations in ribosomal components has highlighted the crucial role of the ribosome in maintaining translational accuracy. Early studies revealed error-prone ribosomal ambiguity in ribosomal protein uS4 in *E. coli* (33), and similar findings were later observed in uS5 in *E. coli* (34). Comparable mutations in uS4 and uS5 have also been documented in *Saccharomyces cerevisiae* (35). In *Mycobacterium smegmatis*, the presence of alternative RP homologs has been shown to result in differential translation efficiency (36). Notably, mutations in uS12 have been associated with enhanced translational fidelity and increased resistance to streptomycin, whereas mutations in uS4 typically lead to reduced translational accuracy (27, 37). Furthermore, ectopic expression of bL20 has been shown to partially recover defects in rRNA processing and 50S biogenesis, suggesting that bL20 may play a coordinated role in proper ribosome assembly, especially under low-temperature conditions (38). Moreover, the shift from translational fidelity to mistranslation appears to be a critical component of the cellular stress response (39, 40).

**Fig 2:**
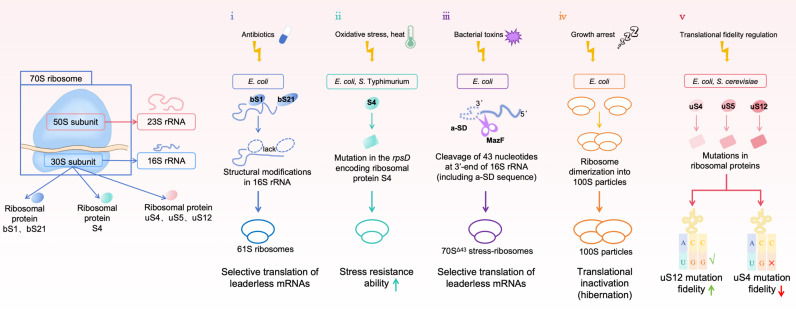
Molecular mechanism of differential compositions of RPs and rRNA to respond to external stress. (ⅰ) Antibiotics promote the formation of 61S ribosomes in *E. coli* both *in vitro* and *in vivo*. The specialized ribosomes lacking several proteins such as bS1 and bS21 exhibit structural modifications in the 16S rRNA and exhibit enhanced ability to selectively translate leaderless mRNAs. (ⅱ) Mutations in *rpsD*, which encodes ribosomal protein S4, have been shown to enhance resistance to oxidative stress and heat in both *E. coli* and *S*. typhimurium. (ⅲ) Under toxins, the unstable antitoxin is degraded, leading to the activation of MazF. The MazF system specifically targets the 16S rRNA, cleaving the 3´-terminal 43 nucleotides and anti-Shine-Dalgarno sequence to form 70S^Δ43^ stress ribosomes, which selectively translate newly generated leaderless mRNAs. (ⅳ) In *E. coli*, when cells cease growth, a portion of the ribosomal population undergoes dimerization, forming 100S ribosomal dimers that are translationally inactive and thought to be in a hibernation state. (ⅴ) *E. coli* and *S. cerevisiae* are prone to mutations in ribosomal proteins uS4 and uS5. Among them, mutations in uS12 are associated with enhanced translational fidelity and increased resistance to streptomycin, while mutations in uS4 usually lead to reduced translational accuracy.

Ribosomal heterogeneity can also be observed at the level of the stabilized ribosome. In *E. coli*, when cells cease growth, a portion of the ribosomal population undergoes dimerization, forming 100S ribosomal dimers that are translationally inactive and thought to be in a hibernation state. This represents another example of reversible bacterial stress management, as these hibernating ribosomes can be disassembled and recycled for new rounds of translation once cellular conditions become favorable again (41). In contrast, *Mycobacterium tuberculosis* ribosomes do not form 100S dimers but instead remain stabilized in the associated 70S form, which does not readily dissociate into the 30S and 50S subunits under hypoxic stress conditions (42). Notably, putative ribosome stabilization factors, including RafS, Rv2632c, and Rv1738, were significantly upregulated during nutrient starvation (43). During periods of nutrient limitation, RsfS binds to the 50S subunit, inhibiting the association of the 30S subunit and blocking protein synthesis. This may facilitate the selective translation of leaderless transcripts (44). The detailed information on the differential composition of ribosomal proteins and RNAs was displayed in Fig. 2 and Table 1.

**TABLE 1:** List of differential composition of ribosomal proteins and RNAs in various bacteria to resist stress

Stress condition	Bacteria	Ribosomal protein/RNA Alteration	Functional change in ribosomes	References
Antibiotics stress	*E. coli*	16S rRNA structural modifications (e.g., formation of 61S ribosome)	Selective translation of leaderless sequence mRNAs	(24, 30)
	*S. T*yphimurium	Mutations in *rpsL* (encodes ribosomal protein S12)	Resistance to or dependence on streptomycin and being restrictive in translation	(27)
Oxidative stress	*E. coli*, *S*. Typhimurium	Mutations in *rpsD* (encodes ribosomal protein S4), mistranslation caused by sRNA DsrA	Enhanced resistance to oxidative stress	(26, 28)
Heat shock	*E. coli*	Mutations in *rpsD* (encodes ribosomal protein S4), mistranslation increasing the RpoH level	Enhanced resistance to heat shock	(29)
Bacterial toxins activation	*E. coli*	Cleavage of 43 nucleotides at 3´-end of 16S rRNA (formation of 70S^Δ43^ stress ribosomes)	Selective translation of newly generated leaderless mRNAs	(31, 32)
Growth arrest	*E. coli*	Ribosome dimerization into 100S particles	Translational inactivation (hibernation); reversible reactivation upon favorable conditions	(41)
Hypoxic stress	*M. tuberculosis*	70S ribosomes remain stable (no dissociation into 30S/50S subunits)	Ribosome stabilization under hypoxia	(42)
Nutrient starvation	*M. tuberculosis*	Upregulation of ribosome stabilization factors (RafS, Rv2632c, Rv1738); RsfS binds to the 50S subunit	Inhibition of 30S association; selective translation of leaderless transcripts	(43, 44)
Translational fidelity regulation	*E. coli*, *S. cerevisiae, S. T*yphimurium	Mutations in uS4, uS5, uS12	Altered fidelity: uS12↑ fidelity (streptomycin resistance); uS4↓ fidelity (stress adaptation)	(26, 33–35, 37)
Low-temperature conditions	*E. coli*	Ectopic expression of bL20	Recovery of rRNA processing and 50S biogenesis defects	(38)

### Post-translational modifications (PTMs) of ribosomal proteins act as precision tools for translational control

**Fig 3:**
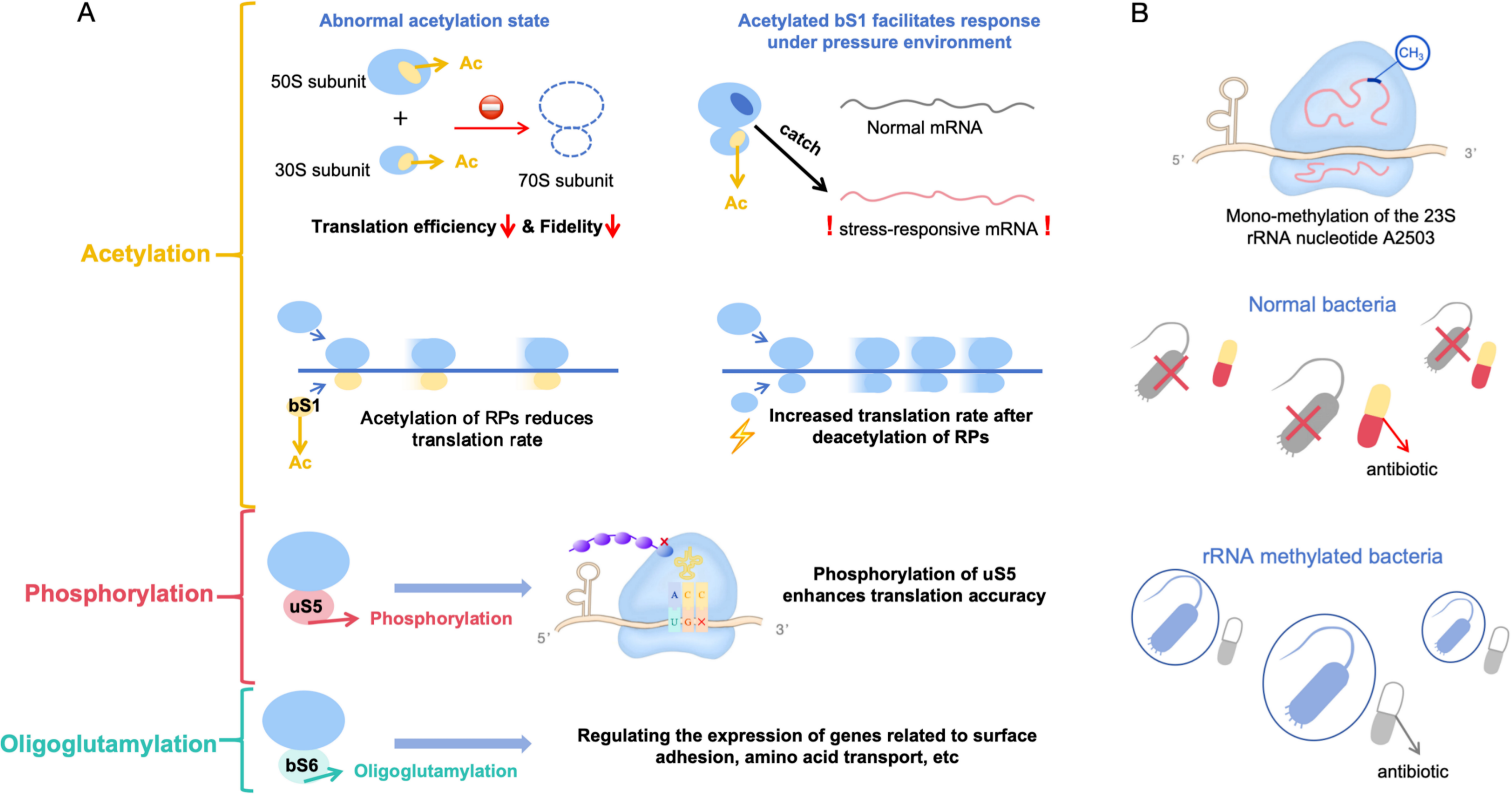
Modification of ribosomal proteins affects protein translation efficiency and methylation of rRNA promotes antibiotic resistance. (**A**) Bacterial RPs are subject to a range of post-translational modifications that influence translation processes. In *E. coli*, acetylation of RPs inhibits the formation of 70S ribosomes and disrupts protein translation. Acetylation driven by acetyl phosphate significantly decreased the relative translation rate, while deacetylation partially restored the translation activity in *E. coli*. Besides, the acetylated bS1 changes its mRNA-binding specificity, enabling selective recruitment of stress-responsive mRNAs to manage nutrient starvation. Phosphorylation in uS5 in *S. cerevisiae* and *Salmonella* modulates translational accuracy *in vivo*. RimK catalyzes the oligoglutamylation in bS6 in *Pseudomonas fluorescens*, which regulates the expression of genes involved in surface attachment, amino acid transporters, and secreted molecules required for adaptation to temperature and nutrients fluctuations. (**B**) The important role of methylation of rRNA in bacterial antibiotic resistance. In bacteria, the lack of essential rRNA methylation leads to reduced translation accuracy and impaired response to metabolites. By acquiring specific RNA methyltransferase genes, bacteria can become resistant to ribosome-targeted antibiotics.

Beyond structural diversity, modifications on RPs provide an additional layer of precisive translational regulation. Recent studies have highlighted that bacterial RPs are subject to a range of PTMs that influence translation processes (45, 46). Protein phosphorylation is commonly employed by cells as a rapid and reversible mechanism to modulate translational efficiency in response to variations in cell physiology, such as nutrient availability (47) or stress conditions (48). For instance, the kinase Ctk1 phosphorylates the RP uS5 at a specific site, Ser238, *in vitro*, and this modification has been linked to the regulation of translational accuracy (49). Maintaining optimal translational fidelity is critical for the virulence and host interactions of *Salmonella* (26). Similarly, Ctk1 modulates translational accuracy *in vivo* through the phosphorylation of a different site, Ser176, in uS5 in *S. cerevisiae* (50). Comprehensive analyses of bacterial protein acetylomes have identified numerous acetylated RPs, suggesting that acetylation plays a role in the feedback regulation of translation (51–54). Proper acetylation is essential for the interaction between elongation factors and polysomes, as well as for regulating ribosome translation efficiency and fidelity. In *E. coli*, acetylation of RPs inhibits the formation of 70S ribosomes and disrupts protein translation (45). Normal acetylation homeostasis of RPs is crucial for ribosome assembly, as well as for maintaining translation efficiency and fidelity. Disruption in this homeostasis, either through hyper- or hypo-acetylation, can lead to defective ribosome assembly, reduced translation efficiency, and increased miscoding rates in *S*. Typhimurium (55). Our previous research demonstrated that acetylation regulates the translation machinery (56). Specifically, acetylation driven by acetyl phosphate significantly decreased the relative translation rate, while deacetylation partially restored the translation activity in *E. coli*. Besides, the acetylated bS1 changes its mRNA-binding specificity, enabling selective recruitment of stress-responsive mRNAs to manage nutrient starvation (57). RimK-mediated oligoglutamylation of bS6 occurs only during the stationary phase in *E. coli* (58), although the exact underlying mechanism remains obscure. Similarly, RimK catalyzes the oligoglutamylation of bS6 in *Pseudomonas fluorescens*, which regulates the expression of genes involved in surface attachment, amino acid transporters, and secreted molecules required for adaptation to temperature and nutrient fluctuations (59, 60). Furthermore, the elevated levels of uS10, regulated by σ^28^-dependent sRNA, in conjunction with NusB, enhance transcriptional antitermination of flagellar operons. This process contributes to increased flagellin protein production, flagella numbers, and overall cell motility (61). Selective translational regulation by bS21 has been reported in *Flavobacterium johnsoniae* (62), and the presence of multiple bS21 homologs in *Francisella tularensis* suggests a more complex role for ribosomes in stress adaptation. As one of the last proteins incorporated during 30S assembly, bS21 is loosely bound to and readily exchangeable among ribosomes. Similar to bS1, its absence in certain ribosomal components leads to intrinsic ribosome heterogeneity, potentially providing a regulatory function. We summarize how exactly RPs were chemically modified to regulate protein translation in Table 2 and Fig. 3A.

**TABLE 2:** List of PTMs of ribosomal proteins acts as precision tools for translational control

Modification type	Bacteria	Affected ribosomal protein	Functional change	References
Phosphorylation	*Salmonella*	uS5 (phosphorylated at Ser238)	Regulates translational accuracy; impacts virulence and host interaction	(26)
Phosphorylation	*S. cerevisiae*	uS5 (phosphorylated at Ser176)	Modulates translational fidelity *in vivo*	(50)
Acetylation	*E. coli, Vibrio parahemolyticus, Spiroplasma eriocheiris, Bacillus thuringiensis*	Multiple RPs (e.g., bS1)	Inhibits 70S ribosome formation, reduces translation efficiency; the acetylated bS1 changes its mRNA-binding specificity, enabling selective recruitment of stress-responsive mRNAs to manage nutrient starvation	(45, 51–54, 57)
Acetylation	*S*. Typhimurium	RP hyper-/hypo-acetylation	Defective ribosome assembly, reduced translation efficiency, increased miscoding	(55)
Oligoglutamylation	*E. coli*	bS6 (RimK-mediated oligoglutamylation)	Occurs only in stationary phase; mechanism unclear	(58)
Oligoglutamylation	*Pseudomonas fluorescens*	bS6 (RimK-mediated oligoglutamylation)	Regulates genes for surface attachment, amino acid transport, and nutrient/temperature adaptation	(59, 60)
σ^28^-dependent sRNA regulation	*E. coli*	uS10 (with NusB)	Enhances transcriptional antitermination of flagellar operons; increases flagellin production and motility	(61)
Selective translation regulation	*F. johnsoniae*	bS21	Ribosomal heterogeneity regulation for stress adaptation	(62)

### Methylation of ribosomal RNAs facilitates the resistance of pathogens to antibiotics

Methylation of rRNA is one of the most widespread chemical modifications observed across all living organisms. This modification is typically localized near ribosomal active sites, either on the surface of 16S rRNA or partially embedded within 23S rRNA, corresponding to specific stages in ribosome assembly during which methylation occurs (63). In bacteria, deficiencies in essential rRNA methylation are associated with reduced translation fidelity, compromised responses to metabolites, and defective processing of 17S rRNA (63, 64).

Ribosomes are a common target for various classes of antibiotics, including aminoglycosides, chloramphenicols, tetracyclines, and macrolides. Resistance to ribosome-targeting antibiotics can arise through modifications of the antibiotic binding sites, such as rRNA or RPs. One mechanism of resistance involves the methylation of bacterial rRNA, typically mediated by the acquisition of specific RNA methyltransferase genes (65–67). For instance, mono-methylation of the 23S rRNA nucleotide A2503 within the peptidyl transferase center can confer resistance to chloramphenicol (68). The methyltransferase enzyme Cfr modifies highly conserved adenosine A2503 in 23S rRNA, thereby conferring resistance to a broad spectrum of ribosome-targeting antibiotics (69). N^6^-methyladenosine at position A2058 (m^6^A2058) of 23S rRNA decreases the binding affinity to macrolide antibiotics, such as erythromycin, resulting in drug resistance of *M. tuberculosis* (70). Furthermore, the researcher found that the lax interaction of *M. tuberculosis* Erm with its rRNA produced a unique methylation pattern and resistance to the ketolide telithromycin (71). Resistance of *Streptomyces fradiae* to the macrolide antibiotic was conferred by single methylations at 23S rRNA nucleotides G748 and A2058 acting in synergy (72). Additionally, m^6^A2058 not only provides cross-resistance to three major antibiotic classes (macrolides, lincosamides, and streptogramin B), but also camouflages bacteria from recognition by the Toll-like receptors, thereby evading the innate immune response and facilitating host infections in gram-positive bacteria like *Staphylococcus aureus* and *Streptococcus pneumoniae* (73, 74). However, a recent study demonstrated that *Staphylococcus aureus* harboring m^6^A2058 ribosomes was outcompeted by cells carrying unmodified ribosomes during infections and showed significantly impaired colonization in the absence of an unmodified counterpart (75). The above findings suggest that m^6^A2058 can cause universal antibiotic resistance in a variety of pathogens, and the competitive advantage conferred by m^6^A2058 ribosomes is evident only under antibiotic selective pressure. Subsequently, further investigation revealed that specific genes involved in host interactions, metabolism, and information processing were disproportionally deregulated in mRNA translation, which was associated with a substantial reduction in translational capacity and fidelity in m^6^A2058 ribosomes (75). These findings highlight a general “inefficient translation” mechanism as a trade-off associated with multidrug-resistant ribosomes (Fig. 3B).

Consistent with the concept that increased phenotypic diversity enhances bacterial survival under certain conditions, mistranslation has been identified as a key feature of heterogeneous ribosomes, contributing to the fitness cost associated with antibiotic stress. Taken together, ribosomal heterogeneity plays a critical role in ribosome assembly, translation efficiency, and fidelity, particularly under stressful conditions.

### Pathogen-specific ribosomes mediate adaptation to host-derived stressors

Accumulating evidence indicates that translational regulation associated with ribosomal composition heterogeneity plays a significant role in biological adaptation (20, 23), particularly benefiting pathogenic microorganisms. This strategy enables pathogens to adjust their translational capacity to meet the specific demands of protein synthesis during physiological infection states. Increasingly, studies show how pathogens harness this astute strategy to counteract lethal pressures, resulting in antibiotic resistance, persistent survival, and the escalation of infections. Ribosomal heterogeneity induced by bS21-2, a specific RP homolog, governs gene expression at the level of protein abundance and positively influences virulence in *F. tularensis* (76, 77). Additionally, an unusual ribosomal component in the large subunit, located near the L1 stalk and associated with an extra helix in the 23S rRNA secondary structure, is actively involved in translation regulation in *Mycobacteria*, which may be related to distinctive properties, such as slow growth (78). The interaction between uL2 and the novel sRNA23 is also implicated in the regulation of pathogenicity in *Streptococcus suis* (79). Ribosomes in *M. tuberculosis* exhibit significant structural heterogeneity, suggesting the existence of subpopulations of ribosomes distinct from canonical ribosomes. These specialized ribosomes may be responsible for the translation of leaderless mRNAs in *M. tuberculosis* (80). Proper homeostasis of RP ensures efficient protein translation and promotes virulence in *S*. Typhimurium (45).

Furthermore, pathogens must adapt to various challenging environments within the host during infection, including nutrition deprivation, acid stress, and oxidative stress. A bacterial m^2^A RNA methyltransferase (RlmN), which targets both rRNA and tRNA, has been shown to regulate the translation of stress-related transcripts in response to reactive oxygen species in *Enterococcus faecalis* (81). The stabilization of 70S monosomes is pivotal under nutrient-starved conditions, as these ribosomes are thought to initiate the translation of leaderless transcripts. The immediate survival of *M. tuberculosis* under nitric oxide stress is likely driven by selective degradation of specific proteins and rapid metabolic adjustments, rather than by transcriptional regulation alone (82). Besides, bacterial pathogens must compete with their hosts for limited metal availability resulting from nutritional immunity of the host which restricts metal bioavailability. Bacteria respond to zinc shortage with inactivation of the Zn-dependent transcriptional repressor Zur and the non-Zn-containing paralogs of ribosomal proteins L31, L33, L36, and S14, participating in cellular Zn homeostasis (83, 84). During zinc deprivation, specialized ribosomes are formed and translationally active, while these ribosomes become inactive when zinc depletion increases (43). The ratio of primary to alternative ribosomal protein S18 in *M. tuberculosis* varies during conditions of zinc deprivation, with an increased production of the alternative protein that gets assembled into alternative ribosomes (85, 86). During conditions of stress, *M. tuberculosis* temporarily relies on alternative initiation mechanisms to sustain protein synthesis, which might be favored by specialized and/or stabilized ribosomes (87). This adaptive mechanism might provide the bacteria with a means to ensure protein production in the zinc-depleted extracellular environment, such as within macrophages.

Recent studies have underscored the fundamental role of ribosomes and the translational machinery in the cellular response to cold stress, with a particular focus on their involvement in the selective translation of cold shock mRNAs at the expense of general protein synthesis (88–90). Following a temperature downshift, the synthesis of the initiation factors IF1 (91), IF2 (92), and IF3 (93) is stimulated, whereas rRNA maturation and ribosome assembly are significantly delayed (94). Intriguingly, the increased level of IF2 does not contribute to translational bias but is important for its participation in the assembly and maturation of ribosomes during cold adaptation (92). Specifically, IF3 was shown to be the main factor responsible for promoting cold shock mRNA translation and inhibiting non-cold shock mRNAs by targeting the early steps of protein synthesis (95). Ribosomes containing the most variable rRNAs, encoded by the *rrnI* operon, can direct the preferential translation of a subset of mRNAs in *Vibrio vulnificus*, enabling the rapid adaptation of bacteria to temperature and nutrient shifts (22). Another essential aspect of translational bias is the inhibition of the translation of non-cold shock mRNAs (96).

Thus, a more complex and dynamically regulated process of pathogenic translation in response to challenges is beginning to emerge, surpassing traditional interpretations.

## DISCUSSION AND OUTLOOK

Bacteria adapt to environmental changes by altering the expression of critical genes that promote growth and survival. Similar to how proteomic diversity enables rapid adaptation without genomic alteration, ribosome heterogeneity plays a crucial role in the preferential translation of particular gene subsets (20, 36). As discussed in this review, we highlight how bacteria utilize ribosome heterogeneity to orchestrate energy allocation and selectively translate desired proteins in response to external pressures.

The ribosome, a highly conserved translation machine, exhibits commonalities across eukaryotes, paleontology, and prokaryotes. Ribosomal proteins, which are RNA-binding proteins with a high content of basic amino acids, particularly lysine residues, are frequent targets of PTMs, contributing to ribosomal heterogeneity. Additionally, rRNAs are targeted by various endoribonucleases, leading to leaderless translation, a key component of the adaptive response in many bacteria. Meanwhile, the rapid advances in deep-sequencing techniques, along with the ribosome profiling methodologies, have enabled comprehensive mapping of ribosomal positions and occupancy on cellular mRNAs in bacteria such as *E. coli*, *S*. Typhimurium, and *Bacillus subtilis*. Researchers have increasingly recognized the crucial importance of translational regulation in bacteria, which presents substantial opportunities for further exploration and practical applications.

### Translational trade-offs: mistranslation as a survival strategy under stress

Under most physiological conditions, bacterial translation is tightly regulated to ensure the accuracy of every step. However, it has been observed that specific types of translational errors can confer a selective advantage to bacteria under stress conditions. The specific triggers for these stress responses through mistranslation, along with the associated trade-offs, remain incompletely understood. Furthermore, it remains unclear why different types of translational errors sometimes induce distinct cellular responses and fitness changes. Current understanding of how translational fidelity influences bacterial pathogens within hosts’ environments is limited, and the underlying molecular mechanisms remain largely unidentified. To address these gaps, future studies are essential to elucidate the mechanisms governing translational error responses and to explore how different types of mistranslation affect the interactions between pathogens and their hosts. Such studies hold significant promise for advancing our understanding of bacterial adaptation and pathogenesis, potentially revealing novel therapeutic targets for combating bacterial infections.

### Therapeutic targeting of ribosomal heterogeneity: combating antimicrobial resistance

The emerging bacterial antimicrobial resistance represents a global challenge for public health. The incredible adaptability of bacterial cells enables them to render drugs ineffective or inactive, thereby complicating the design of new drugs that can effectively target resistant bacteria. A deeper mechanistic understanding of the action of currently used drugs could significantly aid in the rational design of novel compounds effective against drug-resistant pathogens. Many pathogens have the ability to modulate protein translation in response to host-induced stress, and this capability can be exploited to identify new ribosomal targets for drug development. For example, pyrazinamide (PZA), a key tuberculosis drug, is converted to pyrazinoic acid (POA) by pyrazinamidase (encoded by *pncA*), whose loss confers PZA resistance. Notably, bS1 (encoded by *rpsA*) has been validated as a direct target of PZA in *M. tuberculosis*, where its inhibition disrupts trans-translation and leads to bacterial death (97). Trans-translation is essential for freeing scarce ribosomes in nonreplicating organisms, and its inhibition may explain the ability of PZA to eradicate persisting organisms. Further study has demonstrated that conformational mutations at the C-terminus of bS1 abolish the POA binding activity (98). Besides, Chen et al. explored the pyrazinamide resistance mechanism of clinical mutants T370P and W403G in bS1 of *M. tuberculosis* (99). Similarly, mutations in the fourth S1 domain might be involved in altering the RpsA activity, resulting in drug resistance. These molecular mechanisms support the feasibility of targeting ribosomal heterogeneity components for antimicrobial development (100). To further develop such strategies, more detailed structural investigation on the ribosome in pathogens is required to understand the precise mechanisms of translational regulation and the modes of action of various ribosome-targeting antibiotics. A comprehensive understanding of these processes could reveal new therapeutic targets, paving the way for more effective treatments against infections caused by clinically drug-resistant bacteria.

As recent studies have shown that ribosome heterogeneity is involved in the interaction between pathogens and therapeutic drugs, the potential application of ribosome heterogeneity in clinical therapy should be worth looking forward to. Future drug development strategies and studies can be based on high-throughput screening for dormant ribosome-specific inhibitors or small molecules disrupting stress-specific rRNA modifications. Existing drug targets can be optimized, or drug analogs should be modified through mechanistic research. However, this is also full of unknown challenges. We need a higher throughput sequencing technology platform as well as more advanced technology to capture the dynamic change of ribosome heterogeneity.

In conclusion, this reversibility of ribosome heterogeneity introduces a novel paradigm in the regulation of bacterial translation, providing bacterial cells with a dynamic mechanism to fine-tune their proteomes in response to fluctuating environmental conditions. This newly recognized capability enhances our understanding of bacterial adaptation and offers promising avenues for the development of targeted therapeutic strategies.
